# Books and Magazines

**Published:** 1912-09

**Authors:** 


### Books and Magazines.

ESSAY ON HASHEESH. By Victor Robinson; price 50 cents. Medical Review of Reviews, publishers, N. Y., 1912.

This small volume, 12mo, p. 83, is a history of the hemp plant in its several varieties; *cannabis indica,* the species from which is derived the potent narcotic drug being that which is meant by *hasheesh.* The book is beautifully written and is intensely interesting reading. It is exhaustive, and contains many facts and references but little known.

PRACTICAL MEDICINE SERIES; GYNECOLOGY: Vol. IV. Dudley: Bachelle. Year Book Publishers, Chicago, 1912. Price $1.25. The complete set of ten, $10.

We have received Volume IV of this well known series, marking one more step in the year's progress in medicine and surgery. Every doctor who would keep step should buy the set—every year. There's nothing like it published.

